# A case of Ménétriere´s disease treated with the monoclonal antibody cetuximab

**DOI:** 10.1007/s12328-019-00994-8

**Published:** 2019-05-23

**Authors:** Arne Carlsen, Tore Bjørn Grimstad, Lars Normann Karlsen, Ole Jacob Greve, Katrine Brække Norheim, Dordi Lea

**Affiliations:** 1 Gastroenterology Unit, Department of Internal Medicine, Stavanger University Hospital, P.O. Box 8100, 4068 Stavanger, Norway; 2 Department of Clinical Science, Faculty of Medicine, University of Bergen, Bergen, Norway; 3 Department of Radiology, Stavanger University Hospital, Stavanger, Norway; 4 Clinical Immunology Unit, Department of Internal Medicine, Stavanger University Hospital, Stavanger, Norway; 5 Department of Pathology, Stavanger University Hospital, Stavanger, Norway; 6 Department of Clinical Medicine, University of Bergen, Bergen, Norway

**Keywords:** Ménétriere´s disease, Hypoproteinemic hypertrophic gastropathy, Cetuximab, Epidermial growth factor receptor

## Abstract

Ménétriere´s disease is a rare disorder of the body and fundus of the stomach, characterized by a massive proliferation of the foveolar cells and subsequent excess mucous secretion. This results in hypoproteinemia due to loss of serum proteins across the gastric mucosa. The cause of Ménétriere´s disease is unknown, and due to the irreversible and premalignant character of the disorder, the patients affected have been subdued to gastrectomy as the only curable treatment. Epidermial growth factor (EGF) has been implicated in the pathogenesis, a finding that makes the disorder receptive to monoclonal antibody treatment against the EGF receptor. In this case report, we present a 41-year-old woman referred to our emergency department due to dizziness, nausea, and vomiting. A thorough medical investigation, combining clinical history, laboratory investigations, an upper endoscopy with full-thickness snare biopsies, and a CT scan confirmed Ménétriere´s disease, and she was successfully treated with the monoclonal antibody cetuximab.

## Case report

A 41-year-old woman was referred to the ER due to 4 weeks of dizziness, nausea, and vomiting. She had previously been diagnosed with antiphospholipid syndrome in connection with spontaneous abortions, but was otherwise healthy. Two weeks prior to the admission, she had undergone a CT scan of the head and an ultrasound of the deep veins of her lower extremities under the suspicion of thrombosis. This was excluded, and she was then referred to us assuming a diagnosis of benign paroxysmal positional vertigo.

The examination upon admission revealed pedal pitting oedemas, otherwise a normal clinical examination. However, she reported an excessive production of mucous and an involuntarily weight loss, and a complete blood workup disclosed a severe hypoalbuminemia of 18.1 g/L (> 36.0 g/L), with a low total protein of 32 g/L (> 64 g/L), and a hypogammaglobulinemia of 2.3 g/L (> 5.4 g/L). All other blood values were normal.

**Fig. 1:**
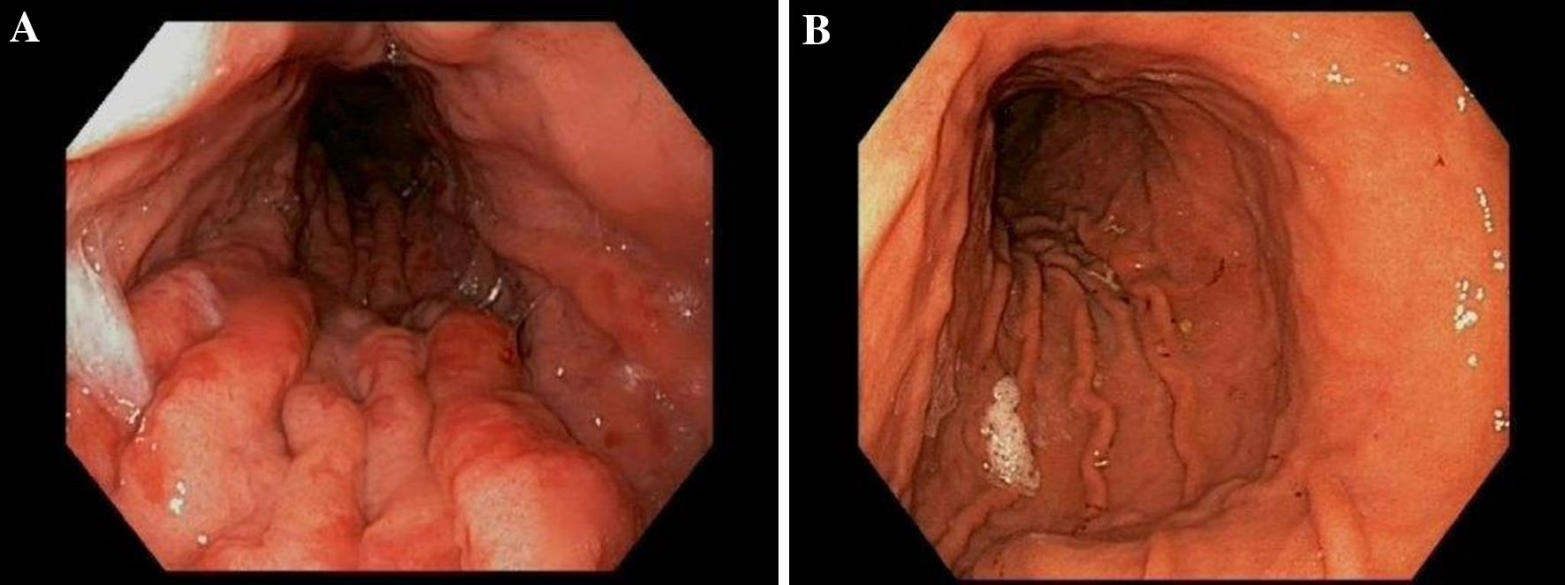
Endoscopy images upon admission (giant hyperemic rugal folds in the body of the stomach, **a**) and at treatment evaluation (normalization of gastric lining, **b**)

**Fig. 2:**
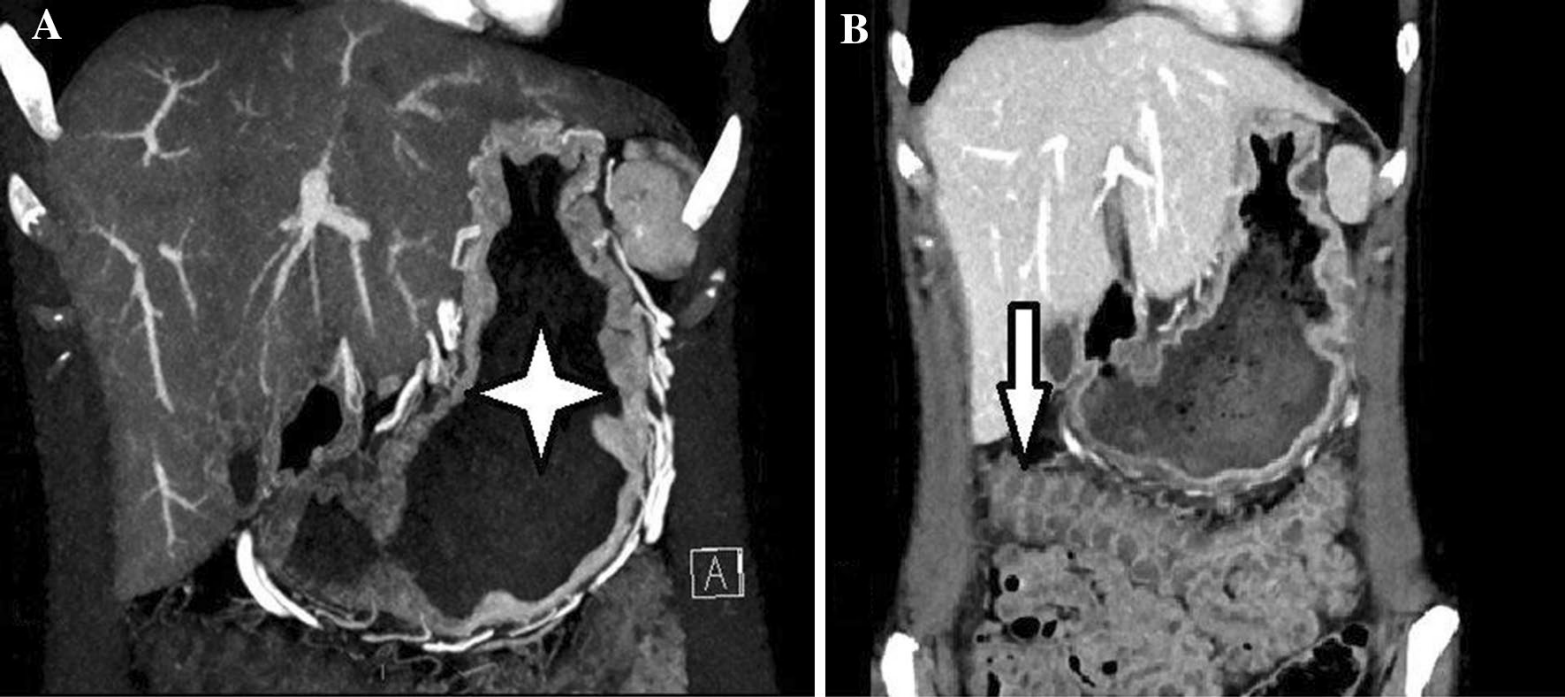
CT angiographic phase with marked hyperemic rugal folds in the body of the stomach (highlighted by star; **a**). Thickened and oedematous colonic haustra project into the lumen of transverse colon in portal venous phase, caused by hypoalbuminemia (highlighted by arrow; **b**)

A gastroscopy was performed, which revealed hyperaemic profoundly enlarged gastric folds in the body of the stomach (Fig. 1a). The histopathological assessment showed a hyperplastic mucous membrane characterized by focal erosions and chronic inflammation, but no signs of malignancy. A contrast-enhanced CT scan of the chest and abdomen showed marked hyperemic rugal folds in the gastric body (Fig. 2a; *star*) as well as hypoalbuminemia-induced thickened and oedematous colonic haustra projected into the transverse colon lumen (Fig. 2b; *arrow*).

**Fig. 3:**
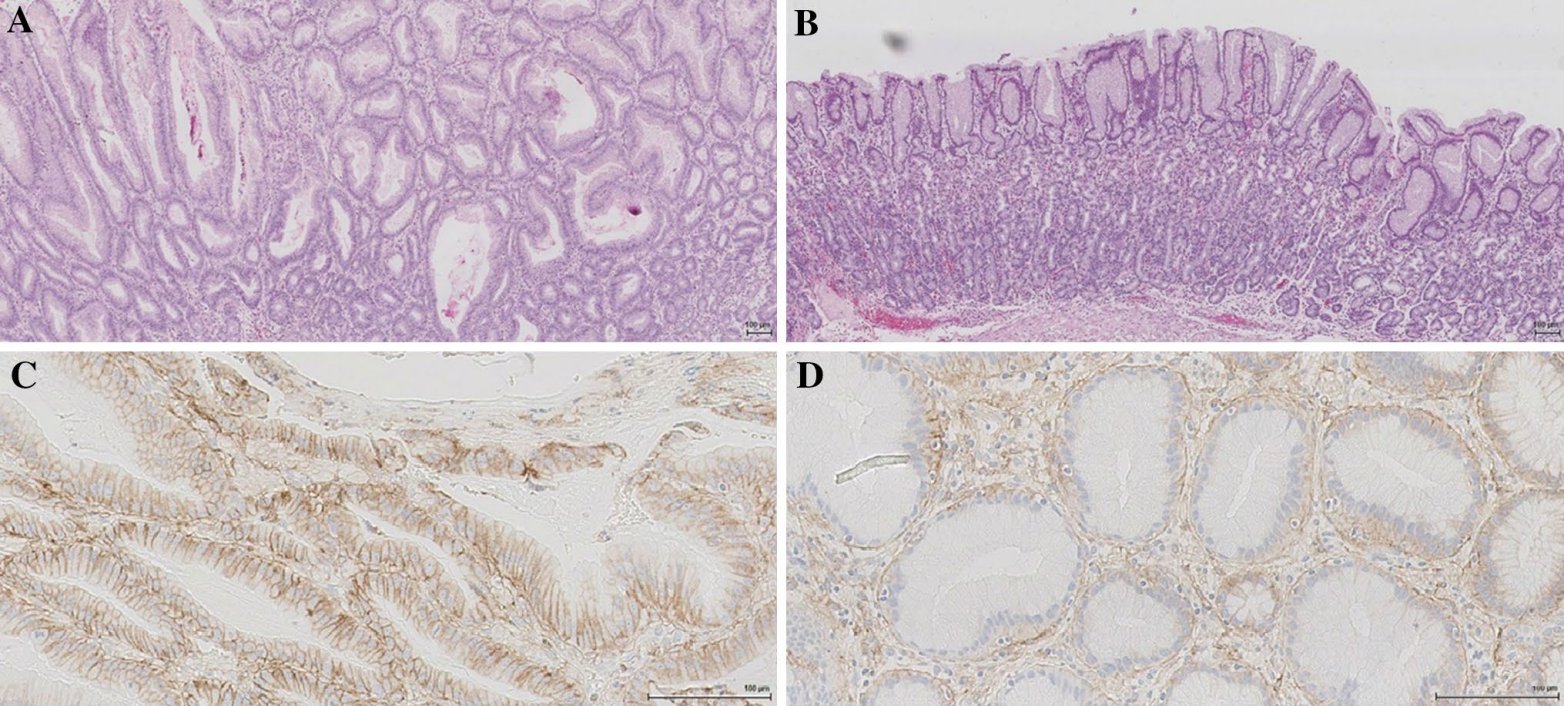
Haematoxylin and Eosin staining of the gastric mucosa before (**a**) and after treatment (**b**) × 40. Immunohistochemical staining of epidermal growth factor receptor (EGFR) 1 shows a membranous staining of the epithelium before treatment (**c**) that is reduced after treatment (**d**) ×200

The CT and endoscopic findings, added to the profound intestinal loss of proteins along with symptoms of mucous emesis, lead to the suspicion of the rare condition Ménétrier’s disease. Infections with cytomegalovirus and Helicobacter pylori were ruled out by serological tests (anti-CMV-IgG positive and anti-CMV-IgM negative, anti-HP-IgG and anti-HP-IgM negative) and by immunohistochemistry (IHC) performed on the biopsies (no staining of inclusions or Helicobacter pylori bacteria). Gastroscopy was repeated and several full-thickness snare biopsies were taken from the body of the stomach. The histopathological examination further supported the diagnosis, with findings of marked foveolar hyperplasia with cystically dilated foveolar glands and prominent mucin production (Fig. 3a).

The patient consented to off-label treatment with cetuximab after a thorough discussion. She was informed of the severity of the disorder, available treatment options, and the prospect of a gastrectomy, but also the sparse documentation of cetuximab (in this setting) and potentially severe adverse events.

This was initiated with a loading dose of 400 mg/m^2^ body surface area followed by weekly infusions of 250 mg mg/m^2^ body surface area [1]. For prevention and treatment of drug side effects, she was administered antibiotics (doxycycline 100 mg bid) and magnesium substitution, as well as a rich moisturizing skin lotion [2].

Her emesis ceased completely after two infusions, and the patient reported a profound and lasting improvement in well being and general quality of life. Blood levels were evaluated before each cetuximab infusion, and total protein, albumin, and gamma globulins in serum were normalized 2 months after treatment initiation. She experienced minor side effects consisting of a nonsevere hypomagnesemia and a mild-to-moderate papulopustular eruption in the face and truncus. The skin manifestations were well controlled after treatment with topical metronidazole and betamethasone [2]. The patient developed fissures in the skin between fingers and toes the first couple of days after each infusion. She was administrated a single infusion of 1000 mg iron(III)isomaltoside due to iron deficiency (ferritin 14 μg/L).

The treatment was evaluated with a gastroscopy after 6 months, which showed complete normalization of the gastric folds and mucosal appearance (Fig. 1b). Full-thickness biopsies of the body of the stomach displayed histopathological normalization of the gastric mucosa **(**Fig. 3b). In addition, there was a reduction of EGFR 1 protein expression with IHC staining after treatment (Fig. 3d) as compared to pre-treatment (Fig. 3c). The treatment was then gradually tapered, and is currently administered once every 6 weeks, with no signs of recurrence of the disease 13 months into follow-up. The treatment interval will be increased to every 12 weeks after 15 months of follow-up.

## Discussion

*Ménétrier’s disease* (hypoproteinemic hypertrophic gastropathy) was first described more than a century ago by Pierre Eugéne Ménétrier, and is characterized by giant rugal folds of the body of the stomach, intestinal protein loss and symptoms including nausea, vomiting, intestinal pain, and peripheral oedemas [3, 4]. Although reversible cases have been described [5, 6], the disease is generally considered irreversible in adults [1, 4, 7]. Ménétrier’s disease has been regarded as a premalignant condition, historically with gastrectomy as the only curable option [7]. Upregulated expressions of transforming growth factor alpha (TGF-α) and epidermal growth factor receptor (EGFR) in the gastric mucosa of individuals diagnosed with Ménétrier’s disease have been demonstrated [8, 9]. A subsequent small single-arm clinical trial with *cetuximab*, a monoclonal antibody that blocks the EGFR signalling, showed promising efficacy and safety. Cetuximab was then proposed as first-line therapy for Ménétrier’s disease [1].

Although treatment duration of this disorder has not been clearly defined, a previous published case series reported treatment periods lasting from 8 to 40 months [1]. In our case report, the patient´s symptoms and endoscopic findings have been evaluated regularly, and cessation of therapy is planned after 24 months. This case report highlights a rare gastrointestinal condition, where the combination of intestinal protein loss and giant gastric folds upon CT scan and/or gastroscopy should lead the clinician’s attention to Ménétrier’s disease. Cetuximab treatment was highly effective in our case, as shown by symptom cessation and normalization of endoscopical, serological as well as in histopathological assessments. The current case report and previously published data support the consideration of cetuximab as first-line treatment in this rare disorder, otherwise often left with gastrectomy as the only treatment option.
